# Prediction of Magnetocaloric Effect Induced by Continuous Modulation of Exchange Interaction: A Monte Carlo Study

**DOI:** 10.3390/ma15217777

**Published:** 2022-11-04

**Authors:** Jiayu Zhang, Jian Wang, Chenyu Zhang, Zongbin Li, Juan Du, Yong Hu

**Affiliations:** 1 Department of Physics, College of Sciences, Northeastern University, Shenyang 110819, China; 2 State Key Laboratory of Rolling and Automation, Northeastern University, Shenyang 110819, China; 3 Key Laboratory for Anisotropy and Texture of Materials (MOE), School of Materials Science and Engineering, Northeastern University, Shenyang 110819, China; 4 Institute of Materials, School of Materials Science and Engineering, Shanghai University, Shanghai 200444, China

**Keywords:** magnetocaloric effect, exchange interaction, Monte Carlo simulation

## Abstract

A magnetic-to-thermal energy conversion, derived from the continuous modulation of intrinsic exchange energy, is conceived and studied by performing Monte Carlo simulations. On the basis of thermodynamics and Weiss’s molecular field theories, we modified the Maxwell formula, where the magnetic entropy change (∆*S*_M_) is calculated by integrating the temperature derivative of magnetization under a continuously increasing exchange interaction, rather than an external magnetic field, from zero to a given value. For the conventional ∆*S*_M_ induced through increasing magnetic field, the ∆*S*_M_ maximum value is enhanced with increasing magnetic field, while the ∆*S*_M_ peak temperature is weakly influenced by the magnetic field. On the contrary, the ∆*S*_M_ induced by changing the exchange interaction is proportional to the exchange interaction while suppressed by a magnetic field. Another feature is that the relative cooling power calculated from the ∆*S*_M_ induced by changing the exchange interaction is fully independent of the magnetic field perspective for obtaining the magnetically stabilized self-converted refrigerants. The controlled variation of exchange interaction could be realized by partial substitution or the application of hydrostatic pressure to lower the cost of magnetic energy at no expense of magnetocaloric response, which opens an avenue to develop the practical and energy-saving devices of conversion from magnetic energy to thermal energy, highly extending the material species of the magnetocaloric effect.

## 1. Introduction

The magnetocaloric effect (MCE) is a magneto-thermodynamic phenomenon, manifested by the changes in isothermal magnetic entropy (∆*S*_M_) and adiabatic temperature (∆*T*_ad_) that accompany magnetic transitions in materials during the application or removal of magnetic field under adiabatic conditions. MCE was first discovered in pure Fe by Emil Warburg in 1881 [1], and independently explained by Debye and Giauque in the 1920s [2,3]. The research interest in MCE has increased in recent decades, on the one hand, due to the possibility of obtaining information about magnetic state and magnetic phase transformations in magnetic materials that is hard to obtain by other methods and, on the other hand, due to the prospects of creation of magnetic cooling machines using magnetic materials as working bodies [4]. Alternative cooling technology methods have been extensively studied owing to the better awareness of the need to identify eco-friendly, cleaner and green technology. In this connection, magnetic refrigeration is proposed as one of the prospective methods. In addition to eco-friendliness, improved Carnot efficiency, compaction and noise minimization are other advantages associated with magnetic cooling [5,6]. All magnetic materials exhibit MCE, although the intensity of this effect depends on the properties of each material. Therefore, extensive research is being carried out to identify suitable magnetic materials for their use as magnetic refrigerants in various temperature ranges.

The measurement methods of MCE can be divided into direct and indirect techniques. In the former, the material is subjected to a magnetic field change and its temperature change is directly measured by some techniques, while in the latter, the MCE is determined on the basis of heat capacity and/or magnetization data. By means of these methods, some breakthroughs in finding MCE materials have been reported in succession and a series of families of MCE materials have been established. Brown in 1968 observed a large ∆*S*_M_ (~10 Jkg^−1^K^−1^) at *T*_C_ = 293 K for Gd [7]. In 1997, Pecharsky and Gschneidner [8] reported that the ∆*S*_M_ of Gd_5_Si_2_Ge_2_ was ~18 Jkg^−1^K^−1^ around *T*_C_ = 278 K for the field change of 0–5 T. In the same year, the ∆*S*_M_ of 5.5 Jkg^−1^K^−1^ for the field change of 1–1.5 T in La_0.8_Ca_0.2_MnO_3_ manganite at *T*_C_ = 274 K was reported [9], followed by the publication in 2000 where Ni_51.5_Mn_22.7_Ga_25.8_ Heusler alloy exhibited a large ∆*S*_M_ of 4.1 Jkg^−1^K^−1^ for the field change of 0–0.9 T associated with the martensitic-to-austenitic phase transition at 197 K [10]. The ∆*S*_M_ maximum values in Gd and La_0.8_Ca_0.2_MnO_3_ manganite appear around the second-order phase transitions which are usually spread over a broad temperature range, beneficial for the active magnetic refrigeration [11,12], while in Gd_5_Si_2_Ge_2_ and Ni_51.5_Mn_22.7_Ga_25.8_ Heusler alloys around the first-order phase transitions which induce a larger ∆*S*_M_ in a narrow temperature range with the harmful thermal/magnetic hysteresis [13].

The relationship between MCE and phase transitions implies the anisotropy and exchange energies responsible for the large MCE besides the external magnetic field, and from the energy-saving point of view, the rotary MCE based on the anisotropy contributing to ∆*S*_M_ has been developed and studied for the aim of reducing the cost of external magnetic field energy [14,15,16,17,18,19,20,21]. On the other hand, effective methods to control the magnetocaloric properties and their working temperatures (probably around the magnetic phase transition temperature) such as partial substitution [22,23], application of hydrostatic pressure [24], and hydrogenation [25] have been proposed. Theoretically, Buchelnikov et al. [26] and Sokolovskiy et al. [27], using Monte Carlo simulations combined with ab initio calculations, studied the micromagnetism and magnetocaloric effect in Co-doped off-stoichiometric Ni-Mn-Ga and Ni-Mn-In Heusler alloys, and the numerical results of magnetic and magnetostructural transitions under a magnetic field agreed fairly well with available experimental data. Although the results may be interpreted by the change of electric structures, metamagnetism and hardening, the separation variation between magnetic moments commonly also changes their exchange forces. Thus we bring a hypothesis: if the exchange interaction in magnetic materials can be tuned continuously, does it (partially) replace the external magnetic field to contribute to the MCE? In this work, the MCE induced by the change of exchange interaction is predicted, which not only highly lowers the external magnetic field under keeping the large MCE to answer the above question *yes*, but also manipulates the large MCE occurring in the room temperature range.

## 2. Model and Monte Carlo Method

**Figure 1:**
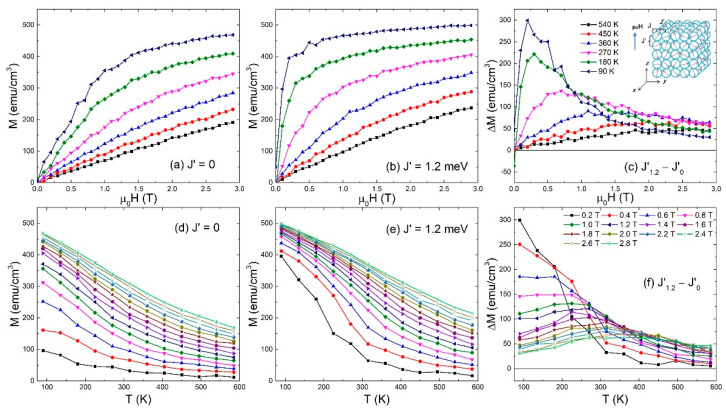
Magnetization for *J*′ = 0 and 1.2 meV and their difference as a function of *μ*_0_*H* at selected *T* and as a function of *T* under selected *μ*_0_*H*. Inset shows the schematic illustration of model with random magnetic anisotropy, where *J*, *J*′, and the magnetic field direction are also labeled.

In the simulation, the 5 × 5 × 5 spins are placed on the node of a simple cubic lattice with periodic boundary conditions. The spins experience random magnetic anisotropy (RMA) and all of them are dipolarly coupled to each other. Moreover, the nearest-neighbor spins are exchange coupled with each other as well. In order to simulate the distinct exchange interactions between different atoms, the exchange interactions (*J*) of spins aligning along with the *x* axis differ from those (*J′*) in the other two orthogonal directions [see inset of Figure 1]. In the presence of a magnetic field, the Hamiltonian of the system can be written as

(1) $$\begin{array}{ll} H= & -\sum_{\underset{x_{i}\neq x_{j}}{<ij>}}J(S_{i}\cdot S_{j})-\sum_{\underset{x_{i}=x_{k}}{<ik>}}J^{\prime }(S_{i}\cdot S_{k})\\ & -\sum_{i}KV{\left(S_{i}\cdot {\hat{e}}_{i}\right)}^{2}+\sum_{i<j}g[\frac{S_{i}\cdot S_{j}}{r^{3}}-3\frac{\left(S_{i}\cdot r)(S_{j}\cdot r\right)}{r^{5}}]\\ & -\sum_{i}NM_{S}V\mu_{0}(S_{i}\cdot H), \end{array}$$

where **S***_i_* denotes the unit vector of spin. The first two terms are the exchange energies, and the exchange constant *J* is fixed as 4 meV to guarantee the occurrence of magnetic phase transition at finite temperature, while *J*′ is changeable from 0 to 4 meV for inducing the MCE. The third term is the anisotropy energy, and by considering the atomic diameter of 3 Å, the volume is calculated as *V* = 14.14 Å^3^ and thus the anisotropy constant *K* = 4 meV/atom [28]. The next term is the long-range dipolar energy, where the dipolar constant *g* = 0.1*J* is set to replace the contribution of the demagnetization field, *r* (**r**) is the dimensionless distance (vector) between the *i*th and *j*th spins (with the direction pointing from the *i*th to *j*th spin). The last term is the Zeeman energy, where **H** is the applied magnetic field, *μ*_0_ is the permeability of vacuum, *M*_S_ = 550 emu/cm^3^ is the saturation magnetization, and *N* = 41 is the factor to be used to guarantee the simulation results with the correct orders of magnitude by establishing a model with a much smaller size for catering to the limited present computational capacity [29,30].

In the framework of thermodynamics, the Gibbs free energy *G* is a function of *T*, pressure *p* and *H*, with the total differential

(2) dG=Vdp−SdT−MdH.

Thus, the internal parameters *S* and *M*, conjugated to the external variables *T* and *H*, can be determined by the following equations of state:

(3a) $$S(T,H,p)=-{\left(\frac{\partial G}{\partial T}\right)}_{H,p},$$

(3b) $$M(T,H,p)=-{\left(\frac{\partial G}{\partial H}\right)}_{T,p}.$$

The famous Maxwell relation,

(4) $$\Delta S_{M}^{H}=\int_{0}^{H_{m}}{\left(\frac{\partial M}{\partial T}\right)}_{H}dH,$$

is obtained from Equations (3a,b). On the other hand, the ferromagnetism and the Curie temperature were explained by Weiss in terms of a huge internal ‘molecular field’ proportional to the magnetization [31,32]. This *virtual* internal field is a useful way of approximating the effect of the interatomic Coulomb interaction in quantum mechanics involving *J* and *J*′. In other words, in the framework of molecular field theory, the spin polarization at low temperature and the magnetization variation with decreasing temperature below Curie temperature also depend on the exchange interaction. Hence it is reasonable to modify Equation (4) into a new type of ∆*S*_M_ which is induced by changing the exchange interaction,

(5) $$\Delta S_{M}^{J}=\int_{0}^{H_{m}^{J}}{\left(\frac{\partial M(T,H,H_{J})}{\partial T}\right)}_{H,H_{J}}dH_{J},$$

where *H*_J_ = *E*_J_/*M*_S_*V* is the exchange field and *H* is nonzero to provide a preferred magnetizing direction. Furthermore, the magnetic cooling efficiency is evaluated by considering the magnitude of ∆*S*_M_ and its full-width at half-maximum (*δT*_FWHM_). It is easy to establish the product of the ∆*S*_M_ maximum and *δT*_FWHM_ as

(6) $$\mathrm{RCP}=-\Delta S_{M}^{\max}\times \delta T_{\mathrm{FWHM}},$$

which stands for the so-called relative cooling power (RCP) [12,13,33]. In order to calculate ∆*S*_M_, the magnetic field can be tuned from *μ*_0_*H* = 0 to 2.9 T, and the temperature can be tuned from *T* = 600 to 90 K. The Monte Carlo Metropolis algorithm is used to update the spin state, and the 10^5^ Monte Carlo steps are performed for thermalization, followed by another 10^5^ Monte Carlo steps to average the magnetization [34].

## 3. Results and Discussion

At first, the magnetization behaviors with increasing *μ*_0_*H* at selected *T* for *J*′ = 0 and 1.2 meV are studied with the results presented in Figure 1a,b. With the increasing *μ*_0_*H*, the magnetization increases monotonically. At high *T*, a roughly linear increase in magnetization with *μ*_0_*H* is observed, while at low *T*, the magnetization increases rapidly under low *μ*_0_*H* and slowly under high *μ*_0_*H*, indicating the occurrence of a magnetic phase transition from superparamagnetic to ferromagnetic state with decreasing *T*. The high-*T* magnetization behaviors for *J*′ = 1.2 meV are similar to those for *J*′ = 0 at the same *T*, while at low *T*, the magnetization for *J*′ = 1.2 meV rapidly increases under lower *μ*_0_*H* and the magnetization under high *μ*_0_*H* is more approaching to the saturation value as compared to *J*′ = 0. In order to highlight the role of *J*′ on the magnetization behavior with *μ*_0_*H* and *T*, the magnetization difference (∆*M*) between *J*′ = 0 and 1.2 meV under the same *μ*_0_*H* is given in Figure 1c. With the decreasing *T*, the maximum value of ∆*M* is enhanced; meanwhile, the ∆*M* peak moves to the lower *μ*_0_*H*, indicating that the large ∆*M* can be obtained by *J*′ at low *T* and intermediate *μ*_0_*H*. On the other hand, the magnetization behaviors for *J*′ = 0 and 1.2 meV as well as ∆*M* with *T* under selected *μ*_0_*H* are shown in Figure 1d–f. The magnetization value for *J*′ = 1.2 meV at low *T* is commonly higher than that for *J*′ = 0 at the same *T*, and thus with decreasing *μ*_0_*H*, the maximum value of ∆*M* increases meanwhile the ∆*M* peak moves to the lower *T*.

**Figure 2:**
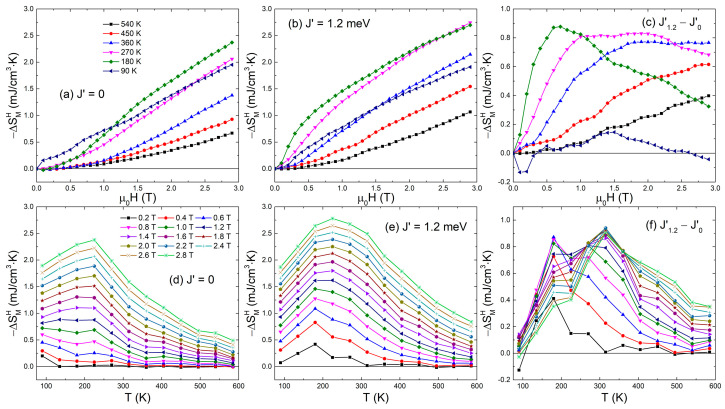
Magnetic entropy change ($-\Delta S_{M}^{H}$) induced by *μ*_0_*H* for *J*′ = 0 and 1.2 meV and their difference as a function of *μ*_0_*H* at selected *T* and as a function of *T* under selected *μ*_0_*H*.

Based on the magnetization results, $-\Delta S_{M}^{H}$ is calculated and its behavior with *μ*_0_*H* and *T* is depicted in Figure 2. With the increasing *μ*_0_*H*, $-\Delta S_{M}^{H}$ increases monotonically at a given *T*. For *J*′ = 0, the largest value of $-\Delta S_{M}^{H}$ under low *μ*_0_*H* (<1 T) is obtained at *T* = 90 K, while under *μ*_0_*H* (>1.1 T) obtained at *T* = 180 K. Similarly, the largest value of $-\Delta S_{M}^{H}$ for *J*′ = 1.2 meV under low *μ*_0_*H* (<2.5 T) is obtained at *T* = 180 K, while under higher *μ*_0_*H* obtained at *T* = 270 K. Furthermore, under the same *μ*_0_*H*, $-\Delta S_{M}^{H}$ for *J*′ = 1.2 meV is commonly larger than that for *J*′ = 0. The difference in $-\Delta S_{M}^{H}$ exhibits a plateau between 0.7 and 0.8 mJ/cm^3^·K with *μ*_0_*H* at *T* = 270 K, while it exhibits a peak at low *T*, e.g., the highest difference of $-\Delta S_{M}^{H}$ (~0.9 mJ/cm^3^·K) is obtained under *μ*_0_*H* = 0.7 T at *T* = 180 K. With the further decreasing *T* down to 90 K, $-\Delta S_{M}^{H}$ for *J*′ = 1.2 meV decreases, and as a result, the difference of $-\Delta S_{M}^{H}$ between *J*′ = 0 and 1.2 meV is smaller than 0.2 mJ/cm^3^·K. On the other hand, the $-\Delta S_{M}^{H}$ behavior with *T* is commonly nonmonotonic under a given *μ*_0_*H*, and with increasing *μ*_0_*H*, the peak of $-\Delta S_{M}^{H}$ moves from *T* = 100 to 225 K for *J*′ = 0 and from *T* = 180 to 225 K for *J*′ = 1.2 meV. At the same *T* and *μ*_0_*H*, $-\Delta S_{M}^{H}$ for *J*′ = 1.2 meV is larger than that for *J*′ = 0, and the high difference of $-\Delta S_{M}^{H}$ appears at *T* = 180 K under low *μ*_0_*H* (≤1 T) and at *T* = 315 K under high *μ*_0_*H* (≥1.4 T). Remarkably, the high *μ*_0_*H* can induce the large $-\Delta S_{M}^{H}$, and the peak of $-\Delta S_{M}^{H}$ commonly appears at room temperature close to the magnetic phase transition temperature, e.g., Curie temperature. In the systems where *J*′ is taken into account, not only the temperature where the peak of $-\Delta S_{M}^{H}$ appears becomes controllable, but also the $-\Delta S_{M}^{H}$ values involving the peak value can be highly enhanced (up to the ~1.3 times enhancement of $-\Delta S_{M}^{H}$ from *J*′ = 0 to 1.2 meV).

**Figure 3:**
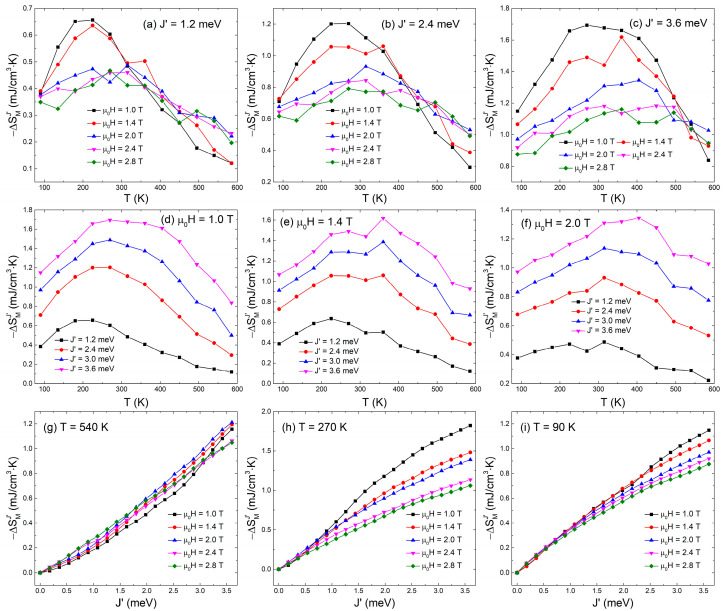
Magnetic entropy change ($-\Delta S_{M}^{J^{\prime }}$) induced by changing *J*′ as a function of *T* at selected *J*′ and *μ*_0_*H* and as a function of *J*′ at selected *T* and *μ*_0_*H*.

Remarkably, the variation of *J*′ induces the magnetization change at given *μ*_0_*H* and *T*, which has been well interpreted by the molecular field theory, and thus Equation (5) should be valid, i.e., the modulation of *J*′ can cause the conversion from magnetic energy to thermal energy via the internal exchange interaction controllable magnetization change. Figure 3 presents the results of $-\Delta S_{M}^{J^{\prime }}$ with *T* and *J*′ under selected *μ*_0_*H*. With the elevating *T*, $-\Delta S_{M}^{J^{\prime }}$ is also nonmonotonic, e.g., for *J*′ = 1.2 meV, the peak value of $-\Delta S_{M}^{J^{\prime }}$ reaches ~0.65 mJ/cm^3^·K under *μ*_0_*H* = 1 T and appears at *T* = 225 K. Interestingly, the strong *μ*_0_*H* can suppress $-\Delta S_{M}^{J^{\prime }}$, opposite to the results of $-\Delta S_{M}^{H}$ vs. *μ*_0_*H*. Moreover, with increasing *J*′, the *T* range of the large $-\Delta S_{M}^{J^{\prime }}$ becomes widened. At a given *T* and *μ*_0_*H*, the larger the *J*′ value is, the larger the $-\Delta S_{M}^{J^{\prime }}$ value that is obtained. The sensitivity of $-\Delta S_{M}^{J^{\prime }}$ with *J*′ to the *μ*_0_*H* depends on *T*, and the highest sensitivity is found near 270 K, as shown in Figure 3. The results indicate that $-\Delta S_{M}^{J^{\prime }}$ is monotonically enhanced by increasing *J*′ while suppressed by applying strong *μ*_0_*H*, and exhibits a peak or a plateau at intermediate *T*, which implies that the energy competition among exchange interaction, magnetic field, and temperature determines the $-\Delta S_{M}^{J^{\prime }}$ behaviors.

**Figure 4:**
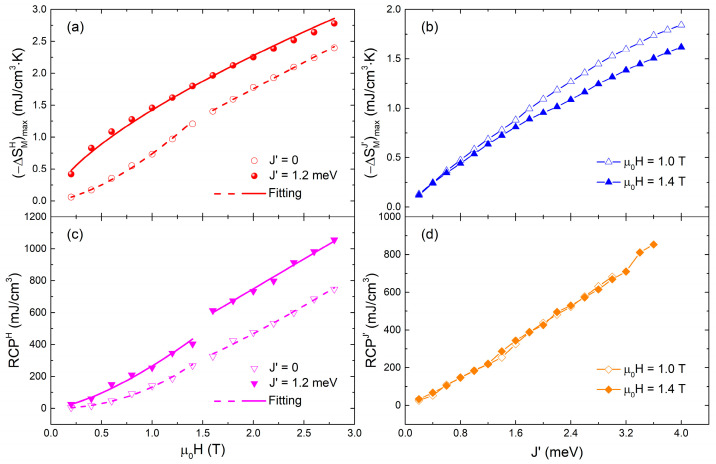
(**a**) Magnetic entropy change maximum value and (**c**) RCP induced by changing *μ*_0_*H* as a function of *μ*_0_*H* at selected *J*′. (**b**) Magnetic entropy change maximum value and (**d**) RCP induced by changing *J*′ as a function of *J*′ under selected *μ*_0_*H*. Solid and open symbols are simulation data results, while solid and dashed curves in (**a**,**c**) are the numerical results fitted by Equations (7) and (8).

Finally, the maximum values of $-\Delta S_{M}^{H}$ with *T* under a given *μ*_0_*H* for *J*′ = 0 and 1.2 meV and the maximum values of $-\Delta S_{M}^{J^{\prime }}$ with *T* at a given *J*′ for *μ*_0_*H* = 1 and 1.4 T are exacted, and the RCP^H^ and RCP^J′^ values are calculated, respectively. As shown in Figure 4, the ${\left(-\Delta S_{M}^{H}\right)}_{\max}$ and RCP^H^ values both increase with the increasing *μ*_0_*H*, and the values obtained for *J*′ = 1.2 meV are larger than those obtained for *J*′ = 0 under the same *μ*_0_*H*. On the other hand, the ${\left(-\Delta S_{M}^{J^{\prime }}\right)}_{\max}$ and RCP^J′^ values both increase with the increasing *J*′. At low *J*′, the values of ${\left(-\Delta S_{M}^{J^{\prime }}\right)}_{\max}$ are approaching under *μ*_0_*H* = 1 and 1.4 T, while at high *J*′, the value of ${\left(-\Delta S_{M}^{J^{\prime }}\right)}_{\max}$ under *μ*_0_*H* = 1 T becomes larger than that under *μ*_0_*H* = 1.4 T. On the contrary, the curves of RCP^J′^ are nearly overlapped under two *μ*_0_*H*, indicating that RCP^J′^ is independent of *μ*_0_*H*. The *μ*_0_*H* dependence of ${\left(-\Delta S_{M}^{H}\right)}_{\max}$ and RCP^H^ can satisfy the power–law relation,

(7) $${\left(-\Delta S_{M}^{H}\right)}_{\max}=a(\mu_{0}H^{n}),$$

(8) $${\mathrm{RCP}}^{H}=b(\mu_{0}H^{m}),$$

where *a* and *b* are constants, the exponent *n* is related to the magnetic order [21,35,36,37], *m* is used to calculate the critical exponent *δ*, related to the *μ*_0_*H* dependence of magnetization during the magnetic phase transition, through the formula

(9) $$m=1+\frac{1}{\delta }.$$

Using *n* and *δ*, the parameter *β*, related to the spontaneous magnetization below the magnetic phase transition temperature, can be obtained by

(10) $$n=1+\frac{1}{\delta }\left[1-\frac{1}{\beta }\right].$$

The results of *n*, *m*, *δ*, and *β* are listed in Table 1. At first, different values of the fit parameters under low and high *μ*_0_*H* are found. The value of *n* is the highest under low *μ*_0_*H* for *J*′ = 0, decreases under high *μ*_0_*H*, and is smaller for *J*′ = 1.2 meV, indicating that the existence of *J*′ favors the magnetic ordering. The value of *δ* is highly enhanced with increasing *μ*_0_*H* for *J*′ = 0, and the value of *δ* under low *μ*_0_*H* for *J*′ = 1.2 meV is as large as that under high *μ*_0_*H* for *J*′ = 0. Therefore, the *μ*_0_*H* and *J*′ both enhance the magnetization change with *μ*_0_*H* during the magnetic phase transition. On the contrary, the value of *β* decreases monotonically from the low *μ*_0_*H* for *J*′ = 0, to the high *μ*_0_*H* for *J*′ = 0, and to the low *μ*_0_*H* for *J*′ = 1.2 meV, designating that the spontaneous magnetizing behavior is easier to occur under high *μ*_0_*H* and at large *J*′. Note that the values of *δ* and *β* under high *μ*_0_*H* for *J*′ = 1.2 meV cannot be obtained, which indicates that a linear response of RCP^H^ to *μ*_0_*H* is achieved under high *μ*_0_*H* at large *J*′.

## 4. Conclusions

In summary, we report a numerical study of the MCE induced by changing *μ*_0_*H* and to predict an intrinsic MCE which is induced by continuously changing the magnetic exchange interaction. The results show that both *μ*_0_*H* and *J*′ can change the magnetization at a given *T*, resulting in nonzero $-\Delta S_{M}^{H}$ and $-\Delta S_{M}^{J^{\prime }}$. The value of $-\Delta S_{M}^{H}$ increases monotonically with increasing *μ*_0_*H*, while exhibits a peak with decreasing *T*. The larger value of *J*′ not only increases the $-\Delta S_{M}^{H}$ value, but also enhances the *T* value where the peak of $-\Delta S_{M}^{H}$ appears. On the other hand, the value of $-\Delta S_{M}^{J^{\prime }}$ increases monotonically with the increasing *J*′, while it decreases with the increasing *μ*_0_*H*. Moreover, the RCP^H^ value increases with the increasing *μ*_0_*H* and *J*′, and the RCP^J′^ value also increases with the increasing *J*′. Interestingly, RCP^J′^ is fully independent of *μ*_0_*H*. The finding is of special importance as the low *μ*_0_*H* range is aimed for in a real household refrigeration scenario as the permanent magnet setup will play the greatest role in price and eco-friendliness, and this intrinsic MCE induced by changing *J*′ not only makes the technology attractive from the high-efficiency and environmental points of view, but also has a considerable scientific interest in itself as a manifestation of the intricate interactions between magnetic degrees of freedom and underlying lattice.

## Figures and Tables

**Table 1:** Calculated critical exponents and parameters of systems without *J*′ and with *J*′ = 1.2 meV under low (0.2~1.4 T) and high (1.6~2.8 T) magnetic field (*μ*_0_*H*).

	*J*′ = 0		*J*′ = 1.2 meV	
	Low *μ*_0_*H*	High *μ*_0_*H*	Low *μ*_0_*H*	High *μ*_0_*H*
*n*	1.5574 ± 0.0270	0.9505 ± 0.0191	0.6779 ± 0.0192	0.6779 ± 0.0192
*m*	2.1546 ± 0.0509	1.4232 ± 0.0643	1.4625 ± 0.0676	0.9994 ± 0.0443
*δ*	0.8661 ± 0.0382	2.3148 ± 0.3590	2.1622 ± 0.3160	−
*β*	1.9334 ± 0.1670	0.8972 ± 0.0499	0.5895 ± 0.0498	−

## Data Availability

The data that support the findings of this study are available from the corresponding author upon reasonable request.
